# On normality, ethnicity, and missing values in quantitative trait locus mapping

**DOI:** 10.1186/1471-2156-6-S1-S52

**Published:** 2005-12-30

**Authors:** Aurélie Labbe, Hanna Wormald

**Affiliations:** 1 Département de Mathématiques et de Statistiques, Université Laval, Québec, G1K7P4, QC, Canada; 2 Department of Statistics and Actuarial Sciences, University of Waterloo, 200 University Avenue West, Waterloo N2L3G1, ON, Canada

## Abstract

**Background:**

This paper deals with the detection of significant linkage for quantitative traits using a variance components approach. Microsatellite markers were obtained for the Genetic Analysis Workshop 14 Collaborative Study on the Genetics of Alcoholism data. Ethnic heterogeneity, highly skewed quantitative measures, and a high rate of missing values are all present in this dataset and well known to impact upon linkage analysis. This makes it a good candidate for investigation.

**Results:**

As expected, we observed a number of changes in LOD scores, especially for chromosomes 1, 7, and 18, along with the three factors studied. A dramatic example of such changes can be found in chromosome 7. Highly significant linkage to one of the quantitative traits became insignificant when a proper normalizing transformation of the trait was used and when analysis was carried out on an ethnically homogeneous subset of the original pedigrees.

**Conclusion:**

In agreement with existing literature, transforming a trait to ensure normality using a Box-Cox transformation is highly recommended in order to avoid false-positive linkages. Furthermore, pedigrees should be sorted by ethnic groups and analyses should be carried out separately. Finally, one should be aware that the inclusion of covariates with a high rate of missing values reduces considerably the number of subjects included in the model. In such a case, the loss in power may be large. Imputation methods are then recommended.

## Background

The purpose of this paper is to illustrate the impact of three factors: robustness to non-normality, population admixture, and covariates with a high rate of missing values, on the linkage detected using a variance components approach. The effect of these factors has been well studied in linkage analysis. As we shall see, the Collaborative Study on the Genetics of Alcoholism (COGA) dataset offers a very good illustration of the dramatic changes observed when such aspects are not considered carefully.

Variance components approaches determine whether genetic variation at a specific chromosomal location can explain the variation in the phenotype [1]. This nonparametric approach is based on the difference of LOD score of the likelihood under the null and alternative hypothesis, where likelihoods are computed under a multivariate normality assumption of the trait under study. This method is known to have optimal power when the model is well specified [2] but is also known as lacking robustness to the normality assumption of the trait [3].

Population admixture is another phenomenon that has been studied [4]. It is known, for example, that the difference in the allele frequencies or disease rates between sub-populations may lead to violation of the key assumptions of Hardy-Weinberg equilibrium (HWE) and linkage equilibrium (LE) between markers. For example, as Grigul et al. [5] showed, linkage results can be greatly affected by clustering families from an admixed population using pedigree-specific marker allele frequencies.

The last aspect considered in this work deals with the inclusion of covariates in the model with a high proportion of missing values. Several methods of imputation for missing values are available, and we refer for example to Fridley et al. [6] regarding the polygenic model. Despite this fact, many genetic analysis packages still ignore individuals with missing covariates in the model. This can have a very strong impact on the results, because the sample size is considerably reduced, affecting the power of the analysis in most cases.

## Methods

Genome-wide scan analysis was performed on 329 microsatellite markers obtained for 1,350 of the 1,614 individuals in the Genetic Analysis Workshop 14 (GAW14) COGA pedigree data.

### Selecting the quantitative traits

We first sought candidate traits that could be used as an illustration of the impact of the factors studied. After performing genome-wide scan analyses on different electrophysiological quantitative phenotypes, we selected three traits, named ttth1, ttdt3, and ttdt4 in the COGA data, that expressed significant linkage in some regions of the genome. These traits are also commonly referred to as event-related potential (ERP) traits.

### Preparing the pedigrees

In order to measure the influence of ethnicity of pedigrees on quantitative trait locus (QTL) linkage detection, we ran a genome-wide scan analysis on two sets of COGA pedigrees. The first set contained all pedigrees as in the GAW14 COGA data (1,614 individuals distributed in 143 families) and the second set contained 105 pedigrees extracted from the initial set of pedigrees, namely those whose members claimed *ethnicity *= 6 (non-Hispanic White, 1,237 individuals). These last pedigrees are called the White pedigrees through this paper. Note that some pedigrees were truncated in order to preserve the unity of the self-reported ethnicity.

### Detecting linkage

All the statistical linkage analyses were carried out using the software SOLAR (Sequential Oligogenic Linkage Analysis Routines) [1,7], which determines whether genetic variation at a specific chromosomal location can explain the variation in the phenotype. This statistical method is an extension of the strategy developed by Amos [8].

Following the variance components method, a quantitative genetic analysis with covariate screening was performed. The best model was chosen by iteratively adding the covariates to the model, and by estimating the different parameters, such as the total additive genetic heritability (H2r) and the covariates regression coefficients, by maximum likelihood. Only significant covariates were kept in the model.

Identity-by-descent (IBD) probabilities and multipoint identity-by-descent (MIBD) matrices (for multipoint analysis) were computed using allele frequencies either provided with the COGA data (not ethnicity-specific) or estimated by SOLAR using a maximum likelihood approach (as in the White pedigree case). In multipoint linkage analysis, the Kosambi map function was used as supplied with the GAW14 data.

### Transformation of the trait to ensure normality

Preparing the quantitative trait for QTL analysis is a crucial step. Normality of the trait is the basic requirement of the statistical method we used. It is important to ensure that the empirical distribution of the trait considered follows this requirement. Skewness and kurtosis are good measures that allow the identification of a potential violation of the normality assumption. Skewness is a measure of the asymmetry of the distribution while kurtosis is an indicator of how close the distribution matches a bell shape. If the distribution is normal, both measures should be zero. A common approach in regression-type models uses the logarithmic transformation. Such a transformation is often effective in reducing skewness and kurtosis. More generally, a Box-Cox transformation (see [9]) can be used in order to maximize the closeness to normality for the transformed data. Data is then transformed to a power λ, where λ is chosen to be optimal. In the case of the COGA dataset, a value λ = 1/4 was found to be optimal for the traits ttth1, ttdt3, and ttdt4, regardless of the type of pedigree used.

### Covariates

If not specified otherwise, the covariates included in the polygenic model (after a screening of all the covariates) involving the transformed trait and the White pedigree were *sex, erpage *(age at ERP examination), *ALDX11 *(alcohol dependency) for trait ttth1^1/4^, *erpage, ALDX12 *for trait ttdt3^1/4 ^and *erpage, ALDX12 *for trait ttdt4^1/4^.

Covariates were taken as provided in the COGA dataset, except in the case of *ALDX1*, *ALDX2 *that were transformed into a number of indicator variables in order to use them as categorical covariates. For instance, *ALDX12 *represents *ALDX1 *= 5 (affected) and *ALDX12 *= 0 represents *ALDX1*<5.

## Results

### Linkage detection

Pedigree-based analyses demonstrated three QTLs on chromosome 1 (between markers D1S1598 and D1S2134; 1p34-1p33), chromosome 18 (between markers D18A535 and D18A877), and chromosome 7 (between markers D7S1804 and D7S509; 7q32-7q34). The first two QTLs appear to be linked to the traits labelled ttdt3 and ttdt4. As mentioned in Table 1, the corresponding LOD scores range from 1.07 up to 4.67, depending on the type of transformation used on the traits as well as the set of pedigrees.

Findings on chromosome 7 show a strong linkage signal for the other measure, ttth1, with a maximum LOD score of 4.08. However, this linkage seems to be strongly related to the self-reported ethnicity of the pedigrees as well as the skewness and kurtosis of the trait.

### Influence of the non-normality of the trait

**Figure 1:**
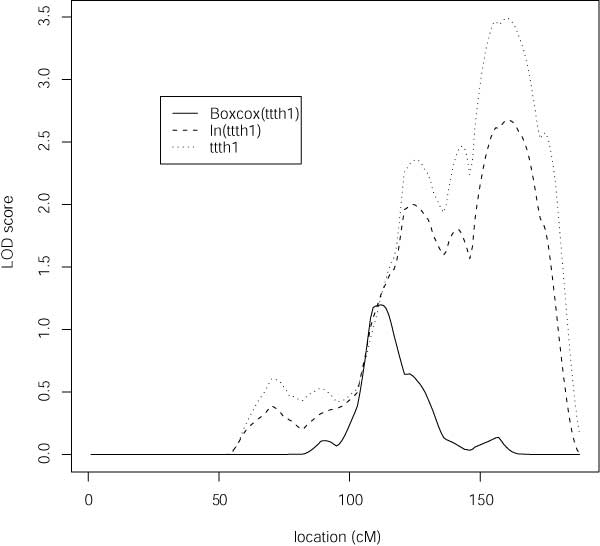
LOD scores for chromosome 7 (White pedigree): transformations of the trait ttth1.

Figure 1 shows the LOD scores across the loci for the traits ttth1, ln(ttth1), and ttth1^1/4^, on chromosome 7 for the White pedigree. Dramatic changes in the LOD scores can be observed when different transformations are applied. The trait ttth1 in its original form appears to be strongly linked to a QTL around 160 cM (LOD = 3.49). The logarithmic transformation of this trait gives only suggestive linkage to the same QTL (LOD = 2.67). However, the Box-Cox transformation of ttth1, i.e., ttth1^1/4^, does not show any linkage result at this locus (LOD = 0.06). A maximum LOD score of 1.19 is obtained at location 112 cM. In this case, not only has the significance of the LOD peak has been dramatically reduced, but it has also been moved by 48 cM.

Clearly, such changes are not systematic across traits, chromosomes, and pedigrees. However, we illustrate here an extreme situation. Table 1 gives information on changes to LOD scores according to the transformation used on traits ttth1, ttdt3, and ttdt4 for chromosomes 1, 7, and 18. This table illustrates a number of different changes in LOD scores when transformations are applied. From this it can be observed that an appropriate transformation on the trait (normality improved) causes changes in the linkage detected (increases of LOD score, as in chromosome 1 and 18, or decreases, as in chromosome 7).

### Influence of ethnicity

Table 1 also illustrates the impact of the segregation of the pedigrees by ethnic groups. Overall linkage findings appeared to be slightly more significant when ethnic admixture is present (note however that the size of population is increased by 30% in this case). Another effect of ethnicity admixture can be noticed in chromosome 7 for trait ttth1. The fact that a QTL is detected and preserved along the transformations in the whole pedigrees only may suggest that it occurs in reality mainly in the non-White pedigrees. Due to the small number of these families available and due to their ethnic heterogeneity, we could not confirm this hypothesis by a statistical analysis.

### Adding more covariates in the polygenic model

**Figure 2:**
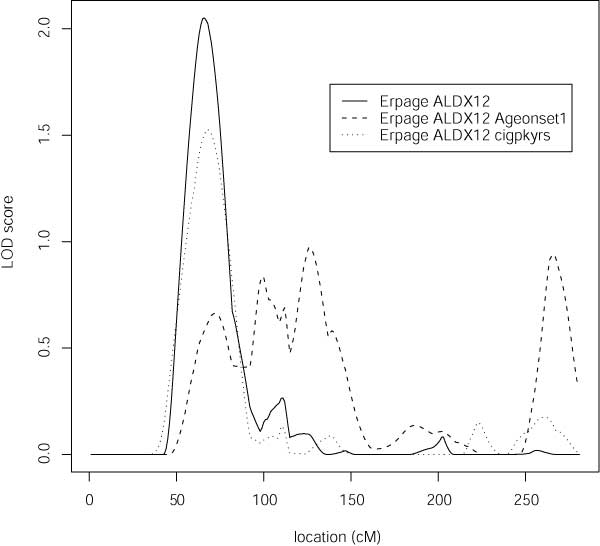
LOD scores for chromosome 1 (White pedigree) using different polygenic models on the trait ttdt3^1/4^.

As an illustration of the impact that the addition of covariates has in the linkage detected, we study the case of chromosome 1 using the White pedigrees and the trait ttdt3^1/4^. Figure 2 presents the changes in LOD scores when different covariates are added in the polygenic model. Note that the optimal model includes two variables, *erpage *and *ALDX12*, with a total of 680 individuals. If the variable *cigpkyrs *(number of packs of cigarettes per day for one year) is also included in the model (for a total of 445 individuals), we observe a deviation from the optimal model, regarding the significance of the LOD score, along the chromosome. If the variable *cigpkyrs *is replaced by the variable *ageonset1*, which contains 59.5% of missing values, the number of subjects included in the model drops to 348 individuals and we can observe extreme changes in the significance of the LOD score.

## Discussion and conclusion

As we have seen, the COGA dataset provides a very clear illustration of the effects that ethnicity, covariates, and the normality of the trait have on the linkage observed. One should be aware that mixing different ethnic groups may introduce some noise, leading to the failure to detect strong linkage that may be present in one or more of the groups. As a result, populations admixtures should be avoided if no evidence suggests that the different ethnic groups behave similarly in terms of the trait and markers considered. Note that in the case studied, the preliminary multivariate analysis of the traits considered showed highly significant segregation by the self-reported *ethnicity*.

When adding covariates in the model, one should pay special attention to the trade-off between the gain of information due to the covariate and the loss of information due to the reduction of the sample size. If possible, imputation methods should be considered.

Finally, any statistical models are built on data assumptions, such as the normality of the trait. Again, being aware of these assumptions and trying to guarantee their validity is key in the success of an analysis. Note that many other factors could be discussed, such as the impact of a violation of some other model assumptions such as Hardy-Weinberg equilibrium and linkage equilibrium. The type of map used is also crucial in fine mapping studies. Some inconsistencies in the maps lead to great differences in the location of the linkage detected, especially when high density SNPs are used.

## Abbreviations

COGA: Collaborative Study on the Genetics of Alcoholism

ERP: Event-related potential

GAW: Genetic Analysis Workshop

HWE: Hardy-Weinberg equilibrium

IBD: Identity by descent

LE: Linkage equilibrium

MIBD: Multipoint identity by descent

QTL: Quantitative trait locus

## Authors' contributions

Both authors contributed equally to this work in all the aspects of the paper.

## Figures and Tables

**Table 1:** QTL LOD score, per pedigree and per type of transformation

Chr	Trait [skewness; kurtosis]		Maximum LOD score and location (cM)			
			
			Whole pedigree		White pedigree	
1	ttdt3	[0.57; 0.25]	1.4	(65 cM)	1.07	(65 cM)
	ln(ttdt3)	[-0.2; 0.083]	2.79	(64 cM)	2.24	(64 cM)
	ttdt3^1/4^	[-0.0006; -0.076]	2.37	(63 cM)	2.03	(65 cM)

1	ttdt4	[0.55; 0.129]	1.14	(67 cM)	1.2	(69 cM)
	ln(ttdt4)	[-0.23; 0.19]	2.29	(65 cM)	2.02	(67 cM)
	ttdt4^1/4^	[-0.01; -0.069]	1.99	(66 cM)	1.86	(66 cM)

18	ttdt3	[0.57; 0.25]	3.63	(54 cM)	3.05	(54 cM)
	ln(ttdt3)	[-0.2; 0.083]	4.2	(54 cM)	4.2	(54 cM)
	tttdt3^1/4^	[-0.0006; -0.076]	4.67	(54 cM)	3.8	(55 cM)

18	ttdt4	[0.55; 0.129]	1.6	(53 cM)	1.69	(55 cM)
	ln(ttdt4)	[-0.23; 0.19]	2.26	(53 cM)	2.26	(53 cM)
	ttdt4^1/4^	[-0.01; -0.069]	2.39	(49 cM)	2.29	(52 cM)

7	ttth1	[0.62; 0.27]	4.08	(154 cM)	3.49	(160 cM)
	ln(ttth1)	[-0.25; 0.55]	3.55	(161 cM)	2.67	(160 cM)
	ttth1^1/4^	[-0.0053; 0.156]	3.87	(161 cM)	1.19	(112 cM)
